# A Model for p38MAPK-Induced Astrocyte Senescence

**DOI:** 10.1371/journal.pone.0125217

**Published:** 2015-05-08

**Authors:** José C. M. Mombach, Bruno Vendrusculo, Cristhian A. Bugs

**Affiliations:** 1 Universidade Federal de Santa Maria, Santa Maria, RS, Brazil; 2 Universidade Federal do Pampa, São Gabriel, RS, Brazil; Cornell University, UNITED STATES

## Abstract

Experimental evidence indicates that aging leads to accumulation of senescent cells in tissues and they develop a secretory phenotype (also known as SASP, for senescence-associated secretory phenotype) that can contribute to chronic inflammation and diseases. Recent results have showed that markers of senescence in astrocytes from aged brains are increased in brains with Alzheimer’s disease. These studies strongly involved the stress kinase p38MAPK in the regulation of the secretory phenotype of astrocytes, yet the molecular mechanisms underlying the onset of senescence and SASP activation remain unclear. In this work, we propose a discrete logical model for astrocyte senescence determined by the level of DNA damage (reparable or irreparable DNA strand breaks) where the kinase p38MAPK plays a central role in the regulation of senescence and SASP. The model produces four alternative stable states: proliferation, transient cycle arrest, apoptosis and senescence (and SASP) computed from its inputs representing DNA damages. Perturbations of the model were performed through gene gain or loss of functions and compared with results concerning cultures of normal and mutant astrocytes showing agreement in most cases. Moreover, the model allows some predictions that remain to be tested experimentally.

## Introduction

Cellular senescence is an anti-tumor program that is triggered by different insults like telomere shortening, oxidative stress and oncogene activation [1–3]. Experimental evidences support that senescent cells accumulate in aging mammal tissues and have an altered phenotype, called SASP (senescence-associated secretory phenotype), that apparently contributes to several aging diseases including Alzheimer’s disease (AD) [3–6]. SASP contributes to ‘inflamm-aging’ (the development of a systemic proinflammatory status with normal aging) which involves an increase of blood plasma levels of inflammatory cytokines like interleukin 6 (IL-6) [7]. AD is an example of inflammaging disease, other cases are atherosclerosis, osteoporosis and diabetes [7]. In the case of AD, astrocyte senescence is claimed to be an important contributor to the development of the pathology [5]. Astrocytes are the most numerous cell type in the human brain and are involved in many essential physiological functions that keep the brain homeostasis, among them the clearance of the Amyloid-β peptide that accumulates in brains with AD [5]. Astrocytes are sensitive to oxidative stress (caused by reactive oxygen species or ROS) which increases with aging and causes DNA damage [8]. The question of whether astrocyte senescence contributes to age-related dementia was recently addressed by Bhat and coworkers who proposed that it is an age-related risk factor for AD [9]. The authors observed *in vitro* that under oxidative stress, astrocytes of brains from patients with AD expressed more senescence and SASP markers than brains from healthy, aged individuals. The chief markers observed include secretion of β-galactosidase, expression of cyclin-dependent kinase inhibitor 2A (p16^INK4a^) and senescence-associated heterochromatin foci [5,10]. The authors verified that astrocytes exposed to Amyloid-β peptides triggered a senescence response and produced high quantities of interleukin 6 (IL-6), a mediator of chronic inflammation that is increased in the central nervous system of AD individuals [5]. In addition, Bath *et al*. observed a strong expression correlation between IL-6 and the mitogen activated protein kinase 14 (p38MAPK) that is an important regulator of cell cycle checkpoints [11,12]. IL-6 in pre-senescent and senescent astrocytes could be abolished by drug inhibition of p38MAPK [9].

These experimental results suggest that astrocyte senescence is strongly connected to p38MAPK activation. However, the exact molecular mechanisms that drive astrocytes into senescence remain obscure [5]. p38MAPK can induce checkpoint arrest and its overexpression induces senescence in fibroblasts which are cells that share functional similarities with astrocytes [5,13]. Based on a previous, specific model of senescence onset at G1/S checkpoint [12], in this work we propose that p38MAPK induction can explain astrocyte senescence and SASP and we propose an extended logical model of the process integrating checkpoints G1/S and G2/M [14] as both have similar mechanisms of checkpoint activation by p38MAPK upon DNA damage [11,15]. The model corroborates several experimental findings and make some predictions.

In what follows we describe the organization of the paper. The logical modeling method is described in the next section. Then after an overview of general molecular mechanisms of checkpoint and cell fate decisions, our model is defined and studied in the Results section. The Discussion section summarizes the implications of this work and indicates future work.

## Methods

Logical models were used to study cell cycle control [16] and cell fate decisions [17], for a review see [14]. A logical model [18–20] is defined by a directed regulatory graph where discrete variables are associated with the nodes and logical rules determine the evolution of these variables. Nodes in this type of graph symbolize molecular components as genes and/or proteins, biological processes (for example, a pathway) or phenomenological events (e.g. apoptosis, senescence etc.). Edges represent activatory or inhibitory effects and variables denote activity levels with two or more states (multi-valued). In most cases the variables are Boolean (0 or 1), but multi-valued variables can represent different influences of a node affecting its targets.

The evolution of the level of each component is defined by a logical rule subjected to the regulators of this component. Input components are not regulated and symbolize extrinsic constant conditions. The dynamics of logical models can be characterized in terms of state transition graphs, where the states are nodes comprising the level of each component in the model and the edges, connecting the nodes, represent state transitions resulting from the logical rules that change the levels of the model components. End nodes in state transition graphs correspond to attractors that can be a stable state (which has no successor state) or a cycle.

The logical framework allows the consideration of diverse molecular processes associated with different time scales in a unique model as it happens with transcriptional regulation and protein phosphorylation [16]. In addition, the logical method permits analysis of perturbations consisting in retaining a variable to its lowest levels, known as loss of function experiment (LoF), or to its positive levels, known as gain of function experiment (GoF).

This framework is implemented in the tool GINsim (http://ginsim.org), which permits different types of analysis of logical models including the determination of stable states [14].

## Results

**Fig 1:**
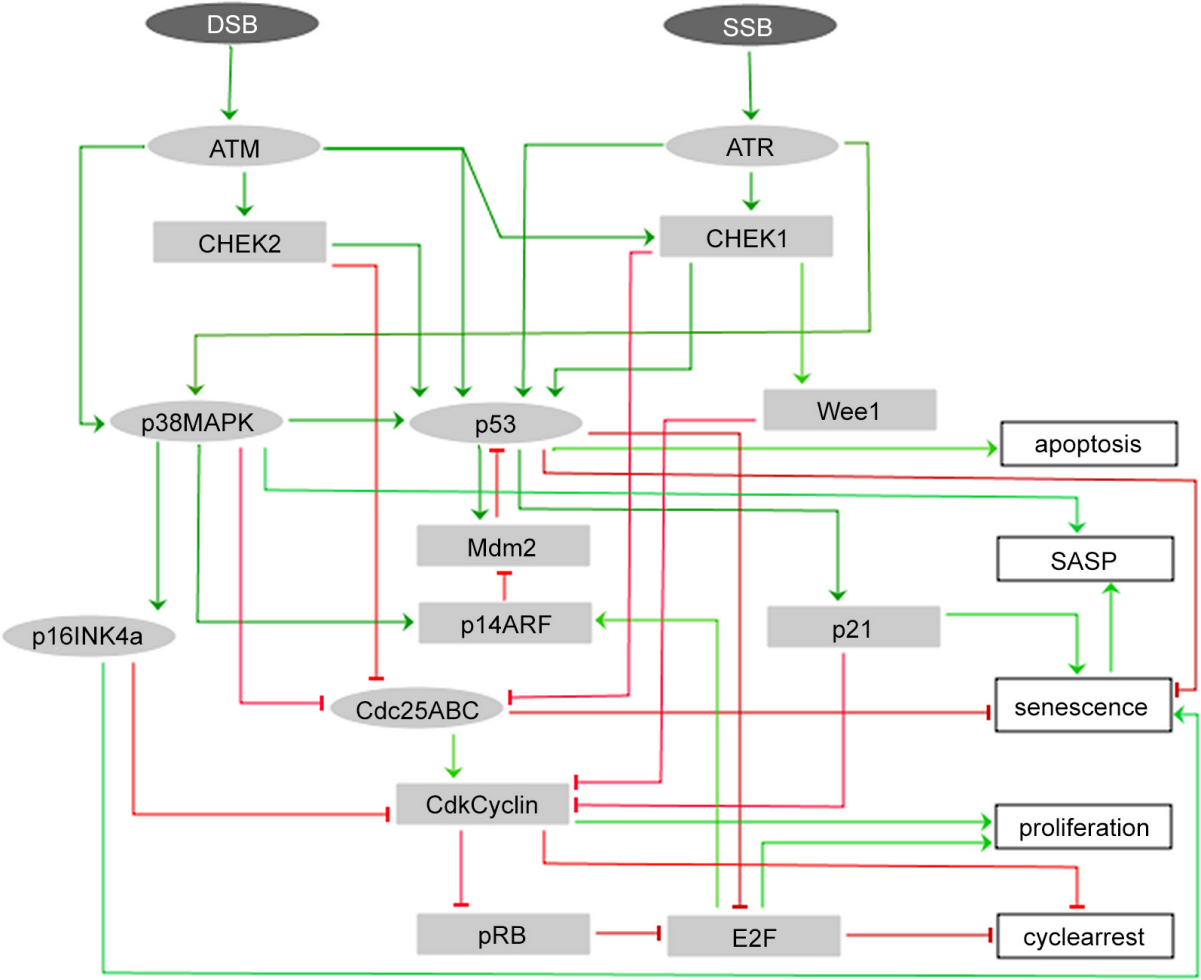
Regulatory network for astrocyte fate decision. Rectangular and elliptic nodes represent Boolean and multi-valued nodes, respectively. The input nodes in dark color at the top of the network denote single (SSB) and double-strand (DSB) DNA breaks, respectively. The output nodes in white color represent the possible cell fate decisions and the internal nodes are the regulators of the outputs.

Cell fate decisions between apoptosis or senescence upon DNA damage occur at cell cycle checkpoints [21]. In what follows, we give an overview of the molecular processes responsible for the induction of cell cycle checkpoints as a result of DNA damage. These responses constitute the focus of the logical regulatory model of Fig 1. Then, we describe our proposal for the mechanisms involved in the regulation of astrocyte senescence and SASP upon checkpoint induction. In a previous work, we introduced a model for the role of p38MAPK on the onset of senescence limited to the G1/S checkpoint [12]. Here, we enlarge this model including the mechanisms activation of the checkpoint G2/M to build a unified framework of checkpoint activation in which p38MAPK regulates the senescence fate [11].

### Checkpoint regulation and apoptosis (Fig 1)

DNA damage activates checkpoints arresting cell cycle progression for a transient arrest for DNA repair or, if the damage is irreparable, a decision is taken between apoptosis or senescence [21,22]. Arrest of the cell cycle can be triggered at G1/S and G2/M checkpoints which have similar molecular mechanisms, in particular, the inhibition of cell division cycle 25 protein family (CDC25A/B/C) required for cell cycle, occurs at both checkpoints.

DNA double-strand breaks activate the kinase ataxia telangiectasia mutated (ATM), either DNA single-strand breaks (SSB) or DSB activate Rad3-related (ATR). Phosphorylations downstream ATM and ATR lead to activation of p53 [22,23]. The cascade phosphorylations triggered by ATM and ATR is shown in Fig 1 [15,21]. The kinase checkpoint kinase 2 (CHEK2) is phosphorylated by ATM while the kinase checkpoint kinase 1 (CHEK1) is phosphorylated by ATR. CHEK2 and CHEK1 start the arrest upregulating Wee1 G2 checkpoint kinase (Wee1) and inactivating CDC25A/B/C required for both checkpoints to activate protein complexes involving cyclins and cyclin-dependent kinases (CDKs) that determine cell cycle progress [15,21]. These complexes are cyclin-dependent kinase 4, 6 and cyclin D (Cdk4/6-Cyclin-D) complex, cyclin-dependent kinase 2 and cyclin E (Cdk2/Cyclin-E) complex for checkpoint G1/S, and cyclin-dependent kinase 1 and cyclin B (Cdk1/Cyclin B) complex (which is inhibited by Wee1) for checkpoint G2/M [21]. In addition, phosphorylated p53 mediates the maintenance of arrest through the activation of cyclin-dependent kinase inhibitor 1A (p21), which also inhibits Cdk4/6-Cyclin-D [24,25]. In the case of checkpoint G1/S, the inhibition of these complexes prevents the phosphorylation of retinoblastoma 1 protein (pRB) and the release of E2F transcription factors that induce the expression of genes required for the cell to enter the S phase [21,26]. In the case of reparable damage, the complexes are reactivated driving the cell to the next phase of the cycle.

E3 ubiquitin protein ligase homolog (Mdm2), p14ARF and p53 form a regulatory circuit. Mdm2 degrades p53 and Mdm2 is sequestered by p14ARF controlling p53 degradation [27].

The choice between cycle arrest and apoptosis occurs through a threshold mechanism dependent on the activation level of p53 that, when exceeded, triggers apoptosis [28]. Owing to this, in our model, apoptosis is activated only when p53 reaches its highest level which is a strong simplification. p14ARF (the alternate reading frame product) and cyclin-dependent kinase inhibitor 2A (p16^INK4a^) contribute to cell cycle regulation and senescence [6,27], deletion of the locus (CDKN2A) that produces these two proteins enhances astrocyte proliferation [29].

### Astrocyte senescence, p38MAPK and SASP (Fig 1)

Experimental results strongly suggest that astrocyte senescence in AD is entangled with the activation of the kinase p38MAPK [9] which, when overexpressed, induces senescence in fibroblasts [5,13,30]. The p38 MAPK family of proteins in which p38α has a prominent role is activated in a ATM/ATR dependent manner by cellular stresses induced, for example, by ROS [8], and it also seems to regulate the secretion of IL-6 in senescent astrocytes [5,9]. IL-6 plays a central role in SASP and inflammaging diseases [3,7].

DNA damage can induce a checkpoint arrest through p38MAPK upon joint mechanisms like: upregulation of p16^INK4a^ and p14ARF, inhibition of the protein family Cdc25A/B/C and phosphorylation of p53 which, additionally, can lead to apoptosis [11,15,31,32].

Senescence requires the activation of p53-p21 and p16^INK4a^-pRB pathways in different cell types. p16^INK4a^ contributes along with p53 to block proliferation as it inhibits cyclin-dependent kinases [6,33,34]. The molecular mechanisms of regulation of p16^INK4a^ (and p14ARF) are not completely understood, but p38MAPK affects the expression of CDKN2A locus [35,36].

### Logical model for astrocyte fate

Based on the biological facts mentioned above, we define a logical model for astrocyte cell cycle checkpoint regulation and fate. The main hypothesis underlying the model is as follows:

In astrocytes senescence activation by p38MAPK upon DNA damage utilizes similar mechanisms for checkpoints G1/S and G2/M.

Tables 1 and 2 include a brief description of the model nodes and of the logical rules governing the states of the nodes, respectively. The logical rules were built based on our interpretation of the biological information, the process also involves several manual rounds of consistency analysis between model predictions and experimental knowledge. The interactions among the nodes in Fig 1 are reported in the literature and detailed bibliographic information can be found in S2 Dataset. Only direct interactions are considered.

**Table 1:** Description of network elements.

Node	Description
SSB	Single strand break: 0 (no break), (1) reparable and (2) irreparable SSB
DSB	Double strand break: 0 (no break), (1) reparable and (2) irreparable DSB
ATR	Ataxia telangiectasia and Rad3 related protein
ATM	Ataxia telangiectasia mutated protein
CHEK2	Checkpoint kinase 2 protein
CHEK1	Checkpoint kinase 1 protein
p14ARF	Alternate reading frame (ARF) protein (from CDKN2A locus)
p16^INK4a^	Cyclin-dependent kinase inhibitor 2A protein (from CDKN2A locus)
p38MAPK	Mitogen activated protein kinase 14 protein
Mdm2	E3 ubiquitin protein ligase homolog protein
p21	Cyclin-dependent kinase inhibitor 1A protein
p53	Tumor supressor protein p53 protein
CDC25ABC	Cell division cycle 25 protein family
E2F	E2F transcription factor family of proteins (E2F1, E2F2, E2F3)
pRB	Retinoblastoma 1 protein
Wee1	Wee1 G2 checkpoint kinase
CdkCyclin	Proteins complexes that promote cell cycle

Short description of the molecular components of the model.

**Table 2:** Logical rules.

Node	Rule / level interpretation	
ATM	1: DSB = 1	Reparable damage
	2: DSB = 2	Persistent DSB signaling
ATR	1: SSB = 1	Reparable damage
	2: SSB = 2	Persistent SSB signaling
CHEK2	1: ATM = 2	
CHEK1	1: ATR = 2 *OR* ATM = 2	
Wee1	1: CHEK1 = 1	
p14ARF	1: p38MAPK = 2 *OR* E2F	
p38MAPK	1: (ATM = 1 *OR* ATR = 1–2) *AND NOT*(ATM = 2)	p38MAPK activation (leading to cycle arrest)
	2: ATM = 2 *AND NOT*(ATR = 2)	p38MAPK activation (leading to senescence)
	3: ATM = 2 *AND* ATR = 2	p38MAPK activation (leading to apoptosis)
Wee1	1: CHEK1	
Mdm2	1: p53 = 1 *AND NOT*(p14ARF)	
p16INK4A	1: p38MAPK = 1–2	Activated p16^INK4A^
	2: p38MAPK = 3	p16 ^INK4A^ upregulation
p21	1: p53 = 1	
p53	1: Mdm2 = 1 *AND* (p38MAPK = 3 *OR* ATR = 1–2 *OR* ATM = 1–2 *OR* CHEK1 = 1 *OR* CHEK2 = 1)	Activated p53 (no accumulation)
	2: *NOT*(Mdm2 = 1) *AND* (p38MAPK = 3 *OR* ATR = 1–2 *OR* ATM = 1–2 *OR* CHEK1 = 1 *OR* CHEK2 = 1)	p53 accumulation leading to apoptosis
Cdc25ABC	1: (p38MAPK = 1–3 *OR* CHEK2 = 1 *OR* CHEK1 = 1) AND NOT(p38MAPK = 1–3 *AND* CHEK2 = 1 *AND* CHEK1 = 1)	Low active Cdc25ABC (non-phosphorylated)
	2: *NOT*(p38MAPK = 1–3) *AND NOT*(CHEK2 = 1) *AND NOT*(CHEK1 = 1)	High concentration of active Cdc25ABC
E2F	1: *NOT*(RB1 = 1) *AND NOT*(p53 = 2)	
pRB	1: *NOT*(CdkCyclin = 1)	Dephosphorylated pRB bound to E2F
CdkCyclin	1: Cdc25A = 1 *AND NOT*(p16INK4a = 1–2) *AND NOT*(p21 = 1) *AND NOT*(Wee1 = 1)	
apoptosis	1: p53 = 2	
SASP	1: p38MAPK *AND* senescence	
proliferation	1: CdkCyclin = 1 *AND* E2F = 1	
senescence	1: (p16INK4a = 1 *AND* p21 = 1 *AND NOT*(Cdc25ABC = 1–2) *AND NOT*(p53 = 2)) *OR* (p16INK4a = 2 *AND* p21 = 1 *AND NOT*(Cdc25ABC = 2) *AND NOT*(p53 = 2))	
cyclearrest	1: *NOT*(CdkCyclin = 1) OR *NOT*(E2F)	

Logical rules controlling the states of the nodes in Fig 1 and interpretation of node levels. The logical operators AND, OR and NOT are used to define the rules for each node defined in terms of the state of its regulators. 0 is the default value. The input components are not shown since they have constant values.

The input nodes of the network, SSB and DSB, can assume three values denoting DNA damage levels: absence of damage = 0, reparable damage = 1 and irreparable damage = 2. SSB and DSB values define ATR and ATM levels, respectively. ATM and ATR activate CHEK2, CHEK1, p38MAPK, Wee1 and p53. DSB can activate CHEK1 through ATM. p53 and p38MAPK are multi-valued and have 3 and 4 levels, respectively, they strongly affect fate decisions. Reparable damage induces p53 to its middle level (p53 = 1) which is involved in several fates. When p53 reaches its highest value 2, apoptosis is triggered but it only occurs for highest DNA damage, *i*.*e*. DSB = SSB = 2 [28]. p38MAPK activation has a stronger influence from ATM than ATR and is controlled in the following way: to reach its first positive level (1) it requires activation of ATR, or ATM but not at its highest level [11]. p38MAPK reaches its level (2) when ATR is not at its maximum level but ATM is. p38MAPK reaches its highest level (3) only when ATM and ATR are both at their maximum levels.

Absence of DNA damage implies the activation of nodes CdkCyclin (representing the protein complexes that promote cell cycle for both checkpoints G1/S and G2/M) and ‘proliferation’. The ‘cycle_arrest’ node represents an arrest for repair and it is inhibited by CdkCyclin and E2F.

The p16^INK4a^-pRB and p53-p21 pathways in astrocytes seem to have redundant function in promoting inhibition of proteins involved in cell cycle progression [37]. Hence, we defined the activation of node ‘senescence’ to require the activation of both, p21 and p16^INK4a^, inactivation of Cdc25ABC and p53 < 2. Nevertheless, if Cdc25ABC is active, senescence can be activated if p16^INK4a^ = 2. SASP requires activation of p38MAPK and senescence [6,9]. Cdc25ABC has 3 levels and can be inactivated only in presence of CHEK1, CHEK2 and p38MAPK [32,38].

In what follows we analyze the model predictions in terms of stable states for the wild-type situation and some selected *in silico* mutations.

### Model results: wild type case

**Fig 2:**
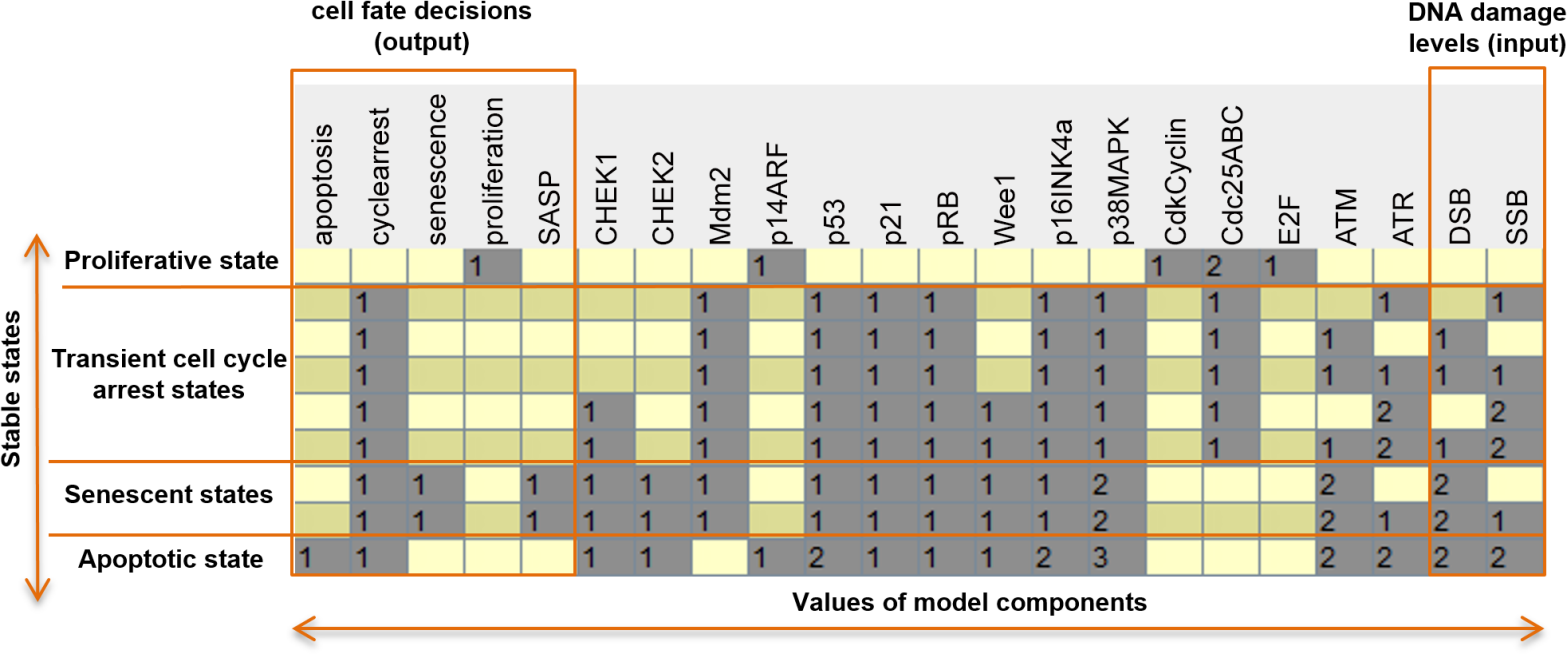
Stable states of the model for astrocyte wild-type case. The two right-most columns list in each line the 9 possible combinations of SSB and DSB. For each line there is a unique stable state characterized by the value of the components and the cell fate is determined by the output components in the 5 left-most columns. Numbers stand for variables state values and empty spaces correspond to state value zero.

This model presents deterministic behavior since each combination of the levels of the input nodes DSB and SSB (nine in total) leads to a unique stable state (see Fig 2) characterized by the activation or deactivation of the nodes representing fates. The synopsis of the results for the wild type case is that no DNA damage (input: DSB = SSB = 0) leads to proliferation, as expected, and the main active components are those involved in cell cycle progression. The highest level of irreparable damage DSB = SSB = 2 yields apoptosis, irreparable DSBs lead to senescence (and SASP) and reparable DSBs result in transient cycle arrest indifferent to the value of SSBs. ‘cycle_arrest’ requires the inhibition of CdkCyclins and it accompanies ‘senescence’ and ‘apoptosis’ which are selected according to the level of DNA damage. DSB = 2 and SSB<2 yield ‘senescence’ which is compatible with the fact that irreparable SSBs seem not able to induce senescence [6,23]. Lastly, ‘apoptosis’ is activated by p53 = 2 when SSB = DSB = 2.

### Comparison of *in silico* mutations with experiments

The model can be perturbed through *in silico* mutations corresponding to loss of function or gain of function experiments. These model perturbations affect the stable states with respect to the wild type case and changes can be related to the predominant growth trend observed in cultures of astrocyte cells undergone LoF or GoF experiments involving one or more genes.

Comparisons of LoF and GoF experiments with model perturbations can be classified in two cases according to DNA damage: (i) absence or (ii) presence of damage that activates checkpoints. DNA damage is assumed to happen in stress situations as seems to be the case of astrocytes in aged brains or in AD and also induced in cultures by ROS, ionizing radiation and other methods.

Only a few experiments with astrocytes were found in the literature and they use primarily mice cells. Given to this lack of information, we report also some interesting perturbations using human or mouse fibroblasts [5,12,13]. Although they will not necessarily yield correct results for astrocytes, they can be considered as model predictions and remain to be verified. Table 3 presents comparisons between model outcomes and LoF and GoF experiments for mutant astrocytes. The full set of stable states of each perturbation is listed in S1 Dataset. In what follows we comment the comparisons in Table 3.

**Table 3:** Comparison with experiments.

**Loss of Function (LoF)**		
**Gene**	**Model outcome (No DNA damage / With DNA damage)**	**Experimental outcome**
p38MAPK	No damage: proliferation / With damage: loss of senescence, apoptosis & SASP	[9]
p16INK4a & p14ARF	No damage: proliferation / With damage: loss of senescence & apoptosis	[29]
p16INK4a	No damage: proliferation / With damage: loss of senescence	[29]
p53	No damage: proliferation / With damage: loss of senescence & apoptosis	[39]
ATM	No damage: proliferation / With damage: loss of senescence & apoptosis	[39,40]
ATR & p53	No damage: proliferation / With damage: loss of senescence & apoptosis	[43]
p21	No damage: proliferation / With damage: loss of senescence	**?**
Cdc25ABC	No damage: cycle arrest / With damage: senescence enhanced	**? /** Fibroblasts: [46]
E2F	No damage: cycle arrest / With damage: similar to the wild type	**? /** Fibroblasts: [47]
pRB	No damage: proliferation / With damage: apoptosis for DSB = SSB = 2 and undetermined for other cases	**? /** Fibroblasts: [48,49]
**Gain of Function (GoF)**		
**Gene**	**Model outcome**	**Experimental outcome**
p38MAPK	No damage: [1–2] cycle arrest; [3] apoptosis / With damage: [1–2] senescence enhanced & loss of apoptosis; [3] apoptosis	**? /** Fibroblasts: [30]
p16INK4a	No damage: cycle arrest / With damage: [1] similar to the wild type; [2] senescence enhanced	**? /** [33]
p53	No damage: [1] cycle arrest; [2] apoptosis / With damage: [1] senescence enhanced; [2] apoptosis	**?**
ATM	No damage: [1] cycle arrest; [2] enhanced senescence / With damage: [1] loss of senescence & apoptosis; [2] senescence enhanced	**?**
p21	No damage: cycle arrest / With damage: similar to the wild type	**? /** Fibroblasts: [44,45]
Cdc25ABC	No damage: proliferation / With damage: [1–2] loss of senescence	**? /** Fibroblasts: [46]
E2F	No damage: proliferation / With damage: apoptosis	**?**
pRB	No damage: cycle arrest / With damage: similar to the wild type	**?**
**LoF + GoF**		
p16INK4a & p14ARF LoF + CdkCyclin GoF	No damage: proliferation / With damage: proliferation	[42]

Comparison of results of perturbations of the model with experiments. Cases for which no experimental data were found are indicated by question marks.

Pharmacological inhibition of p38MAPK in pre-senescent and senescent human astrocytes prevents SASP [9], while the effect of its GoF is yet unknown. However, in human and mouse fibroblasts p38MAPK GoF induces senescence [30]. According to our model p38MAPK LoF abrogates apoptosis, senescence and SASP, while its GoF, with p38MAPK maintained between states 1 and 2, abrogates proliferation and enhances senescence and SASP compatible with fibroblasts experiments. If p38MAPK is maintained at its maximum state 3, then an apoptotic phenotype prevails.

CDKN2A-null mice astrocytes have high proliferation and are used as a model for glioblastoma development [29]. Using the model, this perturbation corresponds to p16INK4a and p14ARF LoF which yields abrogation of senescence and apoptosis which we interpret as compatible with the observed increase of proliferation in experiments.

Given to the importance of p16INK4a in senescence, we studied its perturbations. The model predicts enhanced senescence for GoF of p16INK4a maintained at its state 2 and loss of senescence for its LoF, compatible with observations in other cell types [4].

p53-null mice astrocytes present increased proliferation in culture with small sensitivity to ionizing radiation [39]. The simulation of p53 LoF in absence of DNA damage yields proliferation, in presence of DNA damage it abrogates senescence and apoptosis which should contribute to increased proliferation observed in experiments. GoF of p53 maintained at its state 1 enhances senescence.

ATM-null mice astrocytes show a slight decrease in proliferation in presence or absence of ionizing radiation [39,40]. ATM LoF in the model implies increased proliferation in absence or presence of DNA damage contrasting with experiments. Actually, this is a surprising experimental behavior, in other cell types ATM LoF contributes to senescence suppression [41], however for astrocytes this perturbation seems to have a different effect probably because ATM has some other additional important function in astrocytes that we ignore and that is not contemplated by the present model.

Deletion of CDKN2A and simultaneous overexpression of CDK4 in mice astrocytes generates high proliferating immortal cells and is also studied as a model for glioblastoma development [42]. We simulated a related perturbation with the model by combining the LoF of both p16INK4a and p14ARF with the GoF of CdkCyclin (last line in Table 3). The result is a single output: proliferation, which strongly agrees with the model.

Double mutant ATR;p53-null mice astrocytes show increased proliferation in relation to wild type cultures [43]. For the simulation, the LoF of both ATR and p53 yields proliferation in absence of damage and abrogates senescence and apoptosis compatible with an increase in proliferation.

In what follows we refer to model predictions based on experiments with human or mice fibroblasts, some results are similar to those obtained with our previous model [12].

p21 ectopic expression decreases proliferation and induces senescence in human and mouse fibroblasts [44,45]. For the model, p21 GoF abrogates proliferation in absence of damage agreeing with experiments and its LoF predicts abrogation of senescence.

CDC25ABC LoF and GoF respectively induce or prevent checkpoint arrest in mice fibroblasts [46]. For CDC25ABC LoF, the model enhances senescence and for GoF abrogates senescence agreeing partially with experiments [46].

Human fibroblasts do not proliferate without E2F which agrees with the model that indicates decrease of proliferation with E2F LoF [47]. E2F GoF in human fibroblasts induces apoptosis, however the model only shows this result in presence of DNA damage [47].

pRB-null mice fibroblasts present increased apoptosis [48,49], a phenotype recovered by our model only for the highest damage case. For pRB GoF the model predicts decrease of proliferation in absence of DNA damage.

## Conclusion

Recent experiments suggest that astrocyte senescence (and SASP) is an important component of Alzheimer´s disease [5,9,10]. Motivated by these experiments, in this paper we presented an original model for astrocyte cell fate where p38MAPK plays a central role in the explanation of senescence and SASP induction due to DNA damage [5]. The *in silico* perturbations of the model are consistent with the available experimental data. The model predictions remain to be tested experimentally and one, in particular, the confirmation that p38MAPK GoF in astrocytes induces senescence, if confirmed, would be a strong support for the model [30].

## Supporting Information

S1 Dataset List of model stable states corresponding to *in silico* LoF and GoF perturbations. (PDF)

S2 Dataset Logical model in GINsim file format. Requires GINsim to process it (http://ginsim.org). Bibliographic information about nodes and their interactions are included in the annotations of the algorithm.
(RAR)
