# Supplementary material for: A Model for p38MAPK-Induced Astrocyte Senescence
Source: PLoS One. 2015 May 8;10(5):e0125217. doi: 10.1371/journal.pone.0125217 (PMC4425668; doi:10.1371/journal.pone.0125217)
Supplement: S1 Dataset — (PDF) [file pone.0125217.s001.pdf]

Dataset 1

Paper: Modelling p38MAPK-induced astrocyte senescence

Authors: Bruno Vendrusculo, Cristhian A Bugs, José C.M. Mombach

Model stable states for perturbations corresponding to *in silico* loss of function (LoF) and gain of function (GoF) experiments (see Table 3). Results were obtained using GINsim. For a LoF, the component value is constrained to value 0, whereas for a GoF the value is constrained to value 1 (for a Boolean case), or within a range of positive values (indicated between square brackets). States are displayed in the same way as in Figure 2. Stars (*) denote any damage level. Below each table, a short interpretation indicates the main phenotypic changes compared to the wild type situation.

p38MAPK LoF

apoptosis	cyclearrest	senescence	proliferation	SASP	CHEK1	CHEK2	Mdm2	p14ARF	p53	p21	pRB	Wee1	p16INK4a	p38MAPK	CdkCyclin	Cdc25ABC	E2F	ATM	ATR	DSB	SSB
			1					1							1	2	1				
	1						1		1	1	1					2			1		1
	1						1		1	1	1					2		1		1	
	1						1		1	1	1					2		1	1	1	1
	1				1		1		1	1	1	1				1			2		2
	1				1		1		1	1	1	1				1		1	2	1	2
	1				1	1	1		1	1	1	1				1		2		2	
	1				1	1	1		1	1	1	1				1		2	1	2	1
	1				1	1	1		1	1	1	1				1		2	2	2	2

No DNA damage: proliferation

With DNA damage: loss of senescence, apoptosis & SASP

p38MAPK GoF [1-2]

apoptosis	cyclearrest	senescence	proliferation	SASP	CHEK1	CHEK2	Mdm2	p14ARF	p53	p21	pRB	Wee1	p16INK4a	p38MAPK	CdkCyclin	Cdc25ABC	E2F	ATM	ATR	DSB	SSB
	1										1		1	1		1					
	1						1		1	1	1		1	1		1			1		1
	1						1		1	1	1		1	1		1		1		1	
	1						1		1	1	1		1	1		1		1	1	1	1
	1				1		1		1	1	1	1	1	1		1			2		2
	1				1		1		1	1	1	1	1	1		1		1	2	1	2
	1	1		1	1	1	1		1	1	1	1	1	2				2		2	
	1	1		1	1	1	1		1	1	1	1	1	2				2	1	2	1
	1	1		1	1	1	1		1	1	1	1	1	2				2	2	2	2

No DNA damage: cycle arrest

With DNA damage: senescence enhanced & loss of apoptosis

p38MAPK GoF [3]

apoptosis	cyclearrest	senescence	proliferation	SASP	CHEK1	CHEK2	Mdm2	p14ARF	p53	p21	pRB	Wee1	p16INK4a	p38MAPK	CdkCyclin	Cdc25ABC	E2F	ATM	ATR	DSB	SSB
1	1							1	2	1	1		2	3		1					
1	1							1	2	1	1		2	3		1			1		1
1	1							1	2	1	1		2	3		1		1		1	
1	1							1	2	1	1		2	3		1		1	1	1	1
1	1				1			1	2	1	1	1	2	3		1			2		2
1	1				1			1	2	1	1	1	2	3		1		1	2	1	2
1	1				1	1		1	2	1	1	1	2	3				2		2	
1	1				1	1		1	2	1	1	1	2	3				2	1	2	1
1	1				1	1		1	2	1	1	1	2	3				2	2	2	2

No DNA damage: apoptosis
With DNA damage: apoptosis

p16INK4a & p14ARF LoF

apoptosis	cyclearrest	senescence	proliferation	SASP	CHEK1	CHEK2	Mdm2	p14ARF	p53	p21	pRB	Wee1	p16INK4a	p38MAPK	CdkCyclin	Cdc25ABC	E2F	ATM	ATR	DSB	SSB
			1												1	2	1				
	1						1		1	1	1			1		1			1		1
	1						1		1	1	1			1		1		1		1	
	1						1		1	1	1			1		1		1	1	1	1
	1				1		1		1	1	1	1		1		1			2		2
	1				1		1		1	1	1	1		1		1		1	2	1	2
	1				1	1	1		1	1	1	1		2				2		2	
	1				1	1	1		1	1	1	1		2				2	1	2	1
	1				1	1	1		1	1	1	1		3				2	2	2	2

No DNA damage: proliferation
With DNA damage: loss of senescence & apoptosis

p16INK4a LoF

apoptosis	cyclearrest	senescence	proliferation	SASP	CHEK1	CHEK2	Mdm2	p14ARF	p53	p21	pRB	Wee1	p16INK4a	p38MAPK	CdkCyclin	Cdc25ABC	E2F	ATM	ATR	DSB	SSB
			1					1							1	2	1				
	1						1		1	1	1			1		1			1		1
	1						1		1	1	1			1		1		1		1	
	1						1		1	1	1			1		1		1	1	1	1
	1				1		1		1	1	1	1		1		1			2		2
	1				1		1		1	1	1	1		1		1		1	2	1	2
	1				1	1	1		1	1	1	1		2				2		2	
	1				1	1	1		1	1	1	1		2				2	1	2	1
1	1				1	1		1	2	1	1	1		3				2	2	2	2

No DNA damage: proliferation
With DNA damage: loss of senescence

p16INK4a GoF [1]

apoptosis	cyclearrest	senescence	proliferation	SASP	CHEK1	CHEK2	Mdm2	p14ARF	p53	p21	pRB	Wee1	p16INK4a	p38MAPK	CdkCyclin	Cdc25ABC	E2F	ATM	ATR	DSB	SSB
	1										1		1			2					
	1						1		1	1	1		1	1		1			1		1
	1						1		1	1	1		1	1		1		1		1	
	1						1		1	1	1		1	1		1		1	1	1	1
	1				1		1		1	1	1	1	1	1		1			2		2
	1				1		1		1	1	1	1	1	1		1		1	2	1	2
	1	1		1	1	1	1		1	1	1	1	1	2				2		2	
	1	1		1	1	1	1		1	1	1	1	1	2				2	1	2	1
1	1				1	1		1	2	1	1	1	1	3				2	2	2	2

No DNA damage: cycle arrest
With DNA damage: similar to the wild type

p16INK4a GoF [2]

apoptosis	cyclearrest	senescence	proliferation	SASP	CHEK1	CHEK2	Mdm2	p14ARF	p53	p21	pRB	Wee1	p16INK4a	p38MAPK	CdkCyclin	Cdc25ABC	E2F	ATM	ATR	DSB	SSB
	1										1		2			2					
	1	1					1		1	1	1		2	1		1			1		1
	1	1					1		1	1	1		2	1		1		1		1	
	1	1					1		1	1	1		2	1		1		1	1	1	1
	1	1			1		1		1	1	1	1	2	1		1			2		2
	1	1			1		1		1	1	1	1	2	1		1		1	2	1	2
	1	1		1	1	1	1		1	1	1	1	2	2				2		2	
	1	1		1	1	1	1		1	1	1	1	2	2				2	1	2	1
1	1				1	1		1	2	1	1	1	2	3				2	2	2	2

No DNA damage: cycle arrest
With DNA damage: senescence enhanced

p53 LoF

apoptosis	cyclearrest	senescence	proliferation	SASP	CHEK1	CHEK2	Mdm2	p14ARF	p53	p21	pRB	Wee1	p16INK4a	p38MAPK	CdkCyclin	Cdc25ABC	E2F	ATM	ATR	DSB	SSB
			1					1							1	2	1				
	1										1		1	1		1			1		1
	1										1		1	1		1		1		1	
	1										1		1	1		1		1	1	1	1
	1				1						1	1	1	1		1			2		2
	1				1						1	1	1	1		1		1	2	1	2
	1				1	1					1	1	1	2				2		2	
	1				1	1					1	1	1	2				2	1	2	1
	1				1	1		1			1	1	2	3				2	2	2	2

No DNA damage: proliferation
With DNA damage: loss of senescence & apoptosis

p53 GoF [1]

apoptosis	cyclearrest	senescence	proliferation	SASP	CHEK1	CHEK2	Mdm2	p14ARF	p53	p21	pRB	Wee1	p16INK4a	p38MAPK	CdkCyclin	Cdc25ABC	E2F	ATM	ATR	DSB	SSB
	1						1		1	1	1					2					
	1						1		1	1	1		1	1		1			1		1
	1						1		1	1	1		1	1		1		1		1	
	1						1		1	1	1		1	1		1		1	1	1	1
	1				1		1		1	1	1	1	1	1		1			2		2
	1				1		1		1	1	1	1	1	1		1		1	2	1	2
	1	1		1	1	1		1	1	1	1	1	2	3				2	2	2	2
	1	1		1	1	1	1		1	1	1	1	1	2				2		2	
	1	1		1	1	1	1		1	1	1	1	1	2				2	1	2	1

No DNA damage: cycle arrest
With DNA damage: senescence enhanced

p53 GoF [2]

apoptosis	cyclearrest	senescence	proliferation	SASP	CHEK1	CHEK2	Mdm2	p14ARF	p53	p21	pRB	Wee1	p16INK4a	p38MAPK	CdkCyclin	Cdc25ABC	E2F	ATM	ATR	DSB	SSB
1	1						1		2	1	1					2					
1	1						1		2	1	1		1	1		1			1		1
1	1						1		2	1	1		1	1		1		1		1	
1	1						1		2	1	1		1	1		1		1	1	1	1
1	1				1		1		2	1	1	1	1	1		1			2		2
1	1				1		1		2	1	1	1	1	1		1		1	2	1	2
1	1				1	1		1	2	1	1	1	2	3				2	2	2	2
1	1				1	1	1		2	1	1	1	1	2				2		2	
1	1				1	1	1		2	1	1	1	1	2				2	1	2	1

No DNA damage: apoptosis
With DNA damage: apoptosis

ATM LoF

No DNA damage: proliferation
With DNA damage: loss of senescence & apoptosis

ATM GoF [1]

apoptosis	cyclearrest	senescence	proliferation	SASP	CHEK1	CHEK2	Mdm2	p14ARF	p53	p21	pRB	Wee1	p16INK4a	p38MAPK	CdkCyclin	Cdc25ABC	E2F	ATM	ATR	DSB	SSB
			1					1							1	2	1			*	
	1						1		1	1	1		1	1		1			1	*	1
	1				1		1		1	1	1	1	1	1		1			2	*	2

No DNA damage: cycle arrest
With DNA damage: loss of senescence & apoptosis

ATM GoF [2]

apoptosis	cyclearrest	senescence	proliferation	SASP	CHEK1	CHEK2	Mdm2	p14ARF	p53	p21	pRB	Wee1	p16INK4a	p38MAPK	CdkCyclin	Cdc25ABC	E2F	ATM	ATR	DSB	SSB
	1	1		1	1	1	1		1	1	1	1	1	2				2		*	
	1	1		1	1	1	1		1	1	1	1	1	2				2	1	*	1
1	1				1	1		1	2	1	1	1	2	3				2	2	*	2

No DNA damage: enhanced senescence
With DNA damage: enhanced senescence

ATR & p53 LoF

apoptosis	cyclearrest	senescence	proliferation	SASP	CHEK1	CHEK2	Mdm2	p14ARF	p53	p21	pRB	Wee1	p16INK4a	p38MAPK	CdkCyclin	Cdc25ABC	E2F	ATM	ATR	DSB	SSB
			1					1							1	2	1				*
	1										1		1	1		1		1		1	*
	1				1	1					1	1	1	2				2		2	*

No DNA damage: proliferation
With DNA damage: loss of senescence & apoptosis

p21 LoF

apoptosis	cyclearrest	senescence	proliferation	SASP	CHEK1	CHEK2	Mdm2	p14ARF	p53	p21	pRB	Wee1	p16INK4a	p38MAPK	CdkCyclin	Cdc25ABC	E2F	ATM	ATR	DSB	SSB
			1					1							1	2	1				
	1						1		1		1		1	1		1			1		1
	1						1		1		1		1	1		1		1		1	
	1						1		1		1		1	1		1		1	1	1	1
	1				1		1		1		1	1	1	1		1			2		2
	1				1		1		1		1	1	1	1		1		1	2	1	2
	1				1	1	1		1		1	1	1	2				2		2	
	1				1	1	1		1		1	1	1	2				2	1	2	1
1	1				1	1		1	2		1	1	2	3				2	2	2	2

No DNA damage: proliferation
With DNA damage: loss of senescence

p21 GoF

apoptosis	cyclearrest	senescence	proliferation	SASP	CHEK1	CHEK2	Mdm2	p14ARF	p53	p21	pRB	Wee1	p16INK4a	p38MAPK	CdkCyclin	Cdc25ABC	E2F	ATM	ATR	DSB	SSB
	1									1	1					2					
	1						1		1	1	1		1	1		1			1		1
	1						1		1	1	1		1	1		1		1		1	
	1						1		1	1	1		1	1		1		1	1	1	1
	1				1		1		1	1	1	1	1	1		1			2		2
	1				1		1		1	1	1	1	1	1		1		1	2	1	2
	1	1		1	1	1	1		1	1	1	1	1	2				2		2	
	1	1		1	1	1	1		1	1	1	1	1	2				2	1	2	1
1	1				1	1		1	2	1	1	1	2	3				2	2	2	2

No DNA damage: cycle arrest
With DNA damage: similar to the wild type

Cdc25ABC LoF

apoptosis	cyclearrest	senescence	proliferation	SASP	CHEK1	CHEK2	Mdm2	p14ARF	p53	p21	pRB	Wee1	p16INK4a	p38MAPK	CdkCyclin	Cdc25ABC	E2F	ATM	ATR	DSB	SSB
	1										1										
	1	1					1		1	1	1		1	1					1		1
	1	1					1		1	1	1		1	1				1		1	
	1	1					1		1	1	1		1	1				1	1	1	1
	1	1			1		1		1	1	1	1	1	1					2		2
	1	1			1		1		1	1	1	1	1	1				1	2	1	2
	1	1		1	1	1	1		1	1	1	1	1	2				2		2	
	1	1		1	1	1	1		1	1	1	1	1	2				2	1	2	1
1	1				1	1		1	2	1	1	1	2	3				2	2	2	2

No DNA damage: cycle arrest
With DNA damage: senescence enhanced

Cdc25ABC GoF [1-2]

apoptosis	cyclearrest	senescence	proliferation	SASP	CHEK1	CHEK2	Mdm2	p14ARF	p53	p21	pRB	Wee1	p16INK4a	p38MAPK	CdkCyclin	Cdc25ABC	E2F	ATM	ATR	DSB	SSB
			1					1							1	2	1				
	1						1		1	1	1		1	1		1			1		1
	1						1		1	1	1		1	1		1		1		1	
	1						1		1	1	1		1	1		1		1	1	1	1
	1				1		1		1	1	1	1	1	1		1			2		2
	1				1		1		1	1	1	1	1	1		1		1	2	1	2
	1				1	1	1		1	1	1	1	1	2		1		2		2	
	1				1	1	1		1	1	1	1	1	2		1		2	1	2	1
1	1				1	1		1	2	1	1	1	2	3		1		2	2	2	2

No DNA damage: proliferation
With DNA damage: [1-2] loss of senescence

E2F LoF

apoptosis	cyclearrest	senescence	proliferation	SASP	CHEK1	CHEK2	Mdm2	p14ARF	p53	p21	pRB	Wee1	p16INK4a	p38MAPK	CdkCyclin	Cdc25ABC	E2F	ATM	ATR	DSB	SSB
	1														1	2					
	1						1		1	1	1		1	1		1			1		1
	1						1		1	1	1		1	1		1		1		1	
	1						1		1	1	1		1	1		1		1	1	1	1
	1				1		1		1	1	1	1	1	1		1			2		2
	1				1		1		1	1	1	1	1	1		1		1	2	1	2
	1	1		1	1	1	1		1	1	1	1	1	2				2		2	
	1	1		1	1	1	1		1	1	1	1	1	2				2	1	2	1
1	1				1	1		1	2	1	1	1	2	3				2	2	2	2

No DNA damage: cycle arrest
With DNA damage: similar to the wild type

E2F GoF

apoptosis	cyclearrest	senescence	proliferation	SASP	CHEK1	CHEK2	Mdm2	p14ARF	p53	p21	pRB	Wee1	p16INK4a	p38MAPK	CdkCyclin	Cdc25ABC	E2F	ATM	ATR	DSB	SSB
			1					1							1	2	1				
1	1							1	2	1	1		1	1		1	1		1		1
1	1							1	2	1	1		1	1		1	1	1		1	
1	1							1	2	1	1		1	1		1	1	1	1	1	1
1	1				1			1	2	1	1	1	1	1		1	1		2		2
1	1				1			1	2	1	1	1	1	1		1	1	1	2	1	2
1	1				1	1		1	2	1	1	1	1	2			1	2		2	
1	1				1	1		1	2	1	1	1	1	2			1	2	1	2	1
1	1				1	1		1	2	1	1	1	2	3			1	2	2	2	2

No DNA damage: proliferation
With DNA damage: apoptosis

pRB LoF

apoptosis	cyclearrest	senescence	proliferation	SASP	CHEK1	CHEK2	Mdm2	p14ARF	p53	p21	pRB	Wee1	p16INK4a	p38MAPK	CdkCyclin	Cdc25ABC	E2F	ATM	ATR	DSB	SSB
			1					1							1	2	1				
1	1				1	1		1	2	1		1	2	3				2	2	2	2

No DNA damage: proliferation
With DNA damage: apoptosis for DSB=SSB=2 and undetermined for other cases

pRB GoF

apoptosis	cyclearrest	senescence	proliferation	SASP	CHEK1	CHEK2	Mdm2	p14ARF	p53	p21	pRB	Wee1	p16INK4a	p38MAPK	CdkCyclin	Cdc25ABC	E2F	ATM	ATR	DSB	SSB
	1										1				1	2					
	1						1		1	1	1		1	1		1			1		1
	1						1		1	1	1		1	1		1		1		1	
	1						1		1	1	1		1	1		1		1	1	1	1
	1				1		1		1	1	1	1	1	1		1			2		2
	1				1		1		1	1	1	1	1	1		1		1	2	1	2
	1	1		1	1	1	1		1	1	1	1	1	2				2		2	
	1	1		1	1	1	1		1	1	1	1	1	2				2	1	2	1
1	1				1	1		1	2	1	1	1	2	3				2	2	2	2

No DNA damage: cycle arrest

With DNA damage: similar to the wild type

P16INK4a & p14ARF LoF + CdkCyclin GoF

apoptosis	cyclearrest	senescence	proliferation	SASP	CHEK1	CHEK2	Mdm2	p14ARF	p53	p21	pRB	Wee1	p16INK4a	p38MAPK	CdkCyclin	Cdc25ABC	E2F	ATM	ATR	DSB	SSB
			1												1	2	1				
			1				1		1	1				1	1	1	1		1		1
			1				1		1	1				1	1	1	1	1		1	
			1				1		1	1				1	1	1	1	1	1	1	1
			1		1		1		1	1		1		1	1	1	1		2		2
			1		1		1		1	1		1		1	1	1	1	1	2	1	2
			1		1	1	1		1	1		1		2	1		1	2		2	
			1		1	1	1		1	1		1		2	1		1	2	1	2	1
			1		1	1	1		1	1		1		3	1		1	2	2	2	2

No DNA damage: proliferation

With DNA damage: proliferation
